# Editorial: Highlights in musculoskeletal pain 2021/22

**DOI:** 10.3389/fpain.2023.1139627

**Published:** 2023-02-07

**Authors:** Xiaoxiang Xu

**Affiliations:** Department of Prosthodontics, Peking University School and Hospital of Stomatology, National Center of Stomatology, National Clinical Research Center for Oral Diseases, National Engineering Research Center of Oral Biomaterials and Digital Medical Devices, Beijing Key Laboratory of Digital Stomatology, Research Center of Engineering and Technology for Computerized Dentistry Ministry of Health, NMPA Key Laboratory for Dental Materials, Beijing, China

**Keywords:** chronic musculoskeletal pain, low back pain, microglia, astrocyte, satellite glial cells, spinal cord stimulation

**Editorial on the Research Topic** Highlights in musculoskeletal pain 2021/22

Musculoskeletal pain refers to acute or chronic pain that affects musculoskeletal structures such as bones, muscles, ligaments, tendons, and nerves, which has become the main cause of disability around the world (1). According to the World Health Organization (WHO), there are 1.75 billion people globally with some form of chronic musculoskeletal pain (2). This condition greatly impacts people's life quality and well-being, and creates enormous socio-economic burdens (3). Musculoskeletal pain comprises numerous types, and the prevalence varies. The most common one is low back pain, which affects 30%–40% of adult patients; whilst fibromyalgia only 2% (4). The prevalence of knee pain is 10%–15%, and 15%–20% for neck and shoulder pain (5). Some risk factors have been identified to be associated with musculoskeletal pain, such as smoking, diet, depression, and sedentary lifestyle (6). Though progresses have been made in terms of neural mechanism and management strategy of musculoskeletal pain, challenges still exist especially for the chronic musculoskeletal pain characterized by sustained emotional distress and functional disability. In this special Research Topic *Highlights in Musculoskeletal Pain 2021/22*, we collated a series of articles that provide new knowledge about the epidemiology, mechanism or treatment of musculoskeletal pain.

## Overview of the articles included in this Research Topic

Total twelve articles were collected in this Research Topic: four pieces of original clinical research, three review articles, two original basic studies, one survey report, one hypothesis and one perspective article.

Yalew et al. conducted a cross-sectional investigation among restaurant service staff in Ethiopia to assess the prevalence of work-related low back pain and the associated factors. More than two-fifth of those surveyed reported discomfort in low back area. Several predisposing factors associated were identified such as female, long standing duration while working and carrying out repetitive actions. Recommendations to prevent low back pain for restaurant service staff were provided, including regular exercise and delivering safety training.

In another cross-sectional investigation in India, Sankaran et al. focused on school-going children aged 10–16 from an urban and rural location, exploring the prevalence of musculoskeletal pain among them and its relationship with backpack weight. They reported a high prevalence of musculoskeletal pain in these children, and demonstrated a significant association between backpack weight and musculoskeletal pain.

To study how muscle-muscle interactions act, Dunn et al. tested the modulating effect on hypertonic saline (HS)-induced forearm muscle pain by concurrent infusion of normal saline (NS) into adjacent, contralateral, and remote muscles, that is, the ipsilateral hand, contralateral forearm, and contralateral leg. They showed that subperceptual simultaneous infusion of NS into all these three areas raised the HS-induced overall muscle pain in the forearm. These results implicated the involvement of central nerval system underlying the muscle-muscle interactions.

Administration of non-steroidal anti-inflammatory drugs (NSAIDs) has been noticed to increase the risk of renal complication. Hayashi et al. observed the renal function change of chronic musculoskeletal pain patients with long-term administration of NSAID followed by tramadol hydrochloride/acetaminophen combination tablets (TA). They found that the estimated glomerular filtration rate (eGFR) of patients with NSAIDs administration for 12 months was reduced on cessation of this drug, but there was no reduction of eGFR after TA administration for the following 12 months. This study provides further evidence to highlight the strategy of multimodal analgesic medication against musculoskeletal pain in terms of the potential safety benefit.

Three review articles focused on the role of glia underlying mechanism of nociception. Boakye et al. comprehensively reviewed the process of microglia activation by secondary mediators released from primary afferent neurons, and further the microglia-neuron interaction in the spinal dorsal horn by tertiary mediators released from activated microglia, following peripheral nerve injury in neuropathic pain conditions. They presented an interesting paradox that since many different mediators shared similar effect in the peripheral and central nervous system, how inactivating one mediator can cause the overall pain to be relieved. They also highlighted the different roles of mediators between females and males.

The mini review completed by Gazerani discussed the involvement of peripheral satellite glial cells (SGCs) in pain signaling. The potential future directions in pain research were pointed out by summarizing the promising avenues and the meaningful topics regarding SGCs. Understanding the potential role of SGCs will aid the development of new therapeutics to target pain in the future.

Cedeño et al. reviewed the role of glial cells underlying mechanisms of pain alleviation by spinal cord stimulation using neuropathic pain model in animals. They showed that the approach of differential target multiplexed programming (DTMP) of spinal cord stimulation significantly modulates the transcriptomic profile of neuron and glia cells toward normal levels, indicating a shift in the neuron-glial environment involves in the analgesic effect of spinal cord stimulation.

In their original research article, Ahmed et al. explored whether a gap junction protein (connexin 43) expressed in the trigeminal ganglion is involved in persistent inflammatory hyperalgesia in the temporomandibular joint (TMJ) of both male and female animals. They reported that there was an increased connexin 43 expression following inflammation in TMJ in female rats rather than males. Interestingly, inhibiting connexin 43 in trigeminal ganglion reversed TMJ inflammation-induced masseter muscle overactivity in a sex-independent way, indicating that connexin 43 was involved in the enhancement of jaw muscle activity in both males and females under TMJ inflammation.

In another original research article, Wang et al. explored the effect of c-Jun N-terminal kinase (JNK) on modulating glutamine synthetase (GS) in astrocytes. They observed that GS was activated and phosphorylation of JNK was increased in astrocytes after exposure to lipopolysaccharide (LPS). The changes in GS were reversed following endocannabinoid 2-arachidonoylglycerol (2-AG) administration, but the activation of JNK was not affected, suggesting the phosphorylation of JNK has no effect on modulating of GS in astrocytes by 2-AG.

Clingan et al. presented a brief survey of currently available spinal cord stimulator hardware sold in the United States for the treatment of chronic pain. They introduced the features, indications, and limitations which make each product unique. Understanding each product's nuances will aid the selection of most appropriate device for patients with chronic pain.

In their hypothesis and theory article, Tuckey et al. proposed a novel mechanism of interstitial inflammatory stasis and lymphatic drainage impairment underlying chronic musculoskeletal pain. They hypothesize that inflammatory substance may be entrapped in interstitial space and lymphatic pathways following immune activity or trauma, leading to the interstitial stasis of inflammation. Then the sympathetic mechanism was activated which further decrease blood perfusion and disable the local lymphatic pumping, leading to additional interstitial stasis. This feed-forward loop may play a vital role in the development and maintenance of chronic musculoskeletal pain.

In their perspective article, Schmid et al. come up with a novel cross-disciplinary approach to fill important knowledge gaps in low back pain research, by connecting methods from neuroscience and biomechanics research including functional magnetic resonance imaging, psychological analysis, optical capturing of motion and digital modeling of musculoskeletal system. This novel approach may aid the clarify of motor-control strategy with different phenotypes and the development of better treatment options.
